# Supplementary material for: Transition Metal Coordination Polymers with Trans-1,4-Cyclohexanedicarboxylate: Acidity-Controlled Synthesis, Structures and Properties
Source: Materials (Basel). 2020 Jan 19;13(2):486. doi: 10.3390/ma13020486 (PMC7014088; doi:10.3390/ma13020486)
Supplement: Supplementary file 1 [file materials-13-00486-s001.pdf]

materials

Supplementary

Transition Metal Coordination Polymers with Trans-1,4-Cyclohexanedicarboxylate: Acidity-Controlled Synthesis, Structures and Properties

Pavel A. Demakov [1,2], Artem S. Bogomyakov [2,3], Artem S. Urlukov [1,2], Aleksandra Yu. Andreeva [1], Denis G. Samsonenko [1,2], Danil N. Dybtsev [1,2] and Vladimir P. Fedin [1,2,*]

[1] Nikolaev Institute of Inorganic Chemistry SB RAS, Novosibirsk 630090, Russia; demakov@niic.nsc.ru (P.A.D.); a.urlukov@g.nsu.ru (A.S.U.); andreeva@niic.nsc.ru (A.Y.A.); denis@niic.nsc.ru (D.G.S.); dan@niic.nsc.ru (D.N.D.)

[2] Department of Natural Sciences, Novosibirsk State University, Novosibirsk 630090, Russia; bus@tomo.nsc.ru

[3] International Tomography Center SB RAS, Novosibirsk 630090, Russia

* Correspondence: cluster@niic.nsc.ru

Received: 26 December 2019; Accepted: 17 January 2020; Published: 19 January 2020

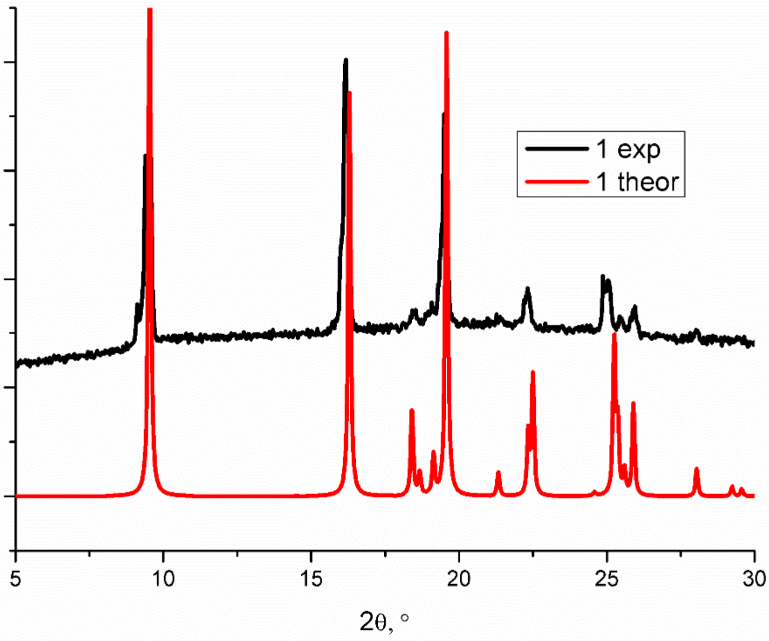

Figure S1. PXRD pattern of the synthesized sample of **1** (black) in comparison with the theoretical one (red).

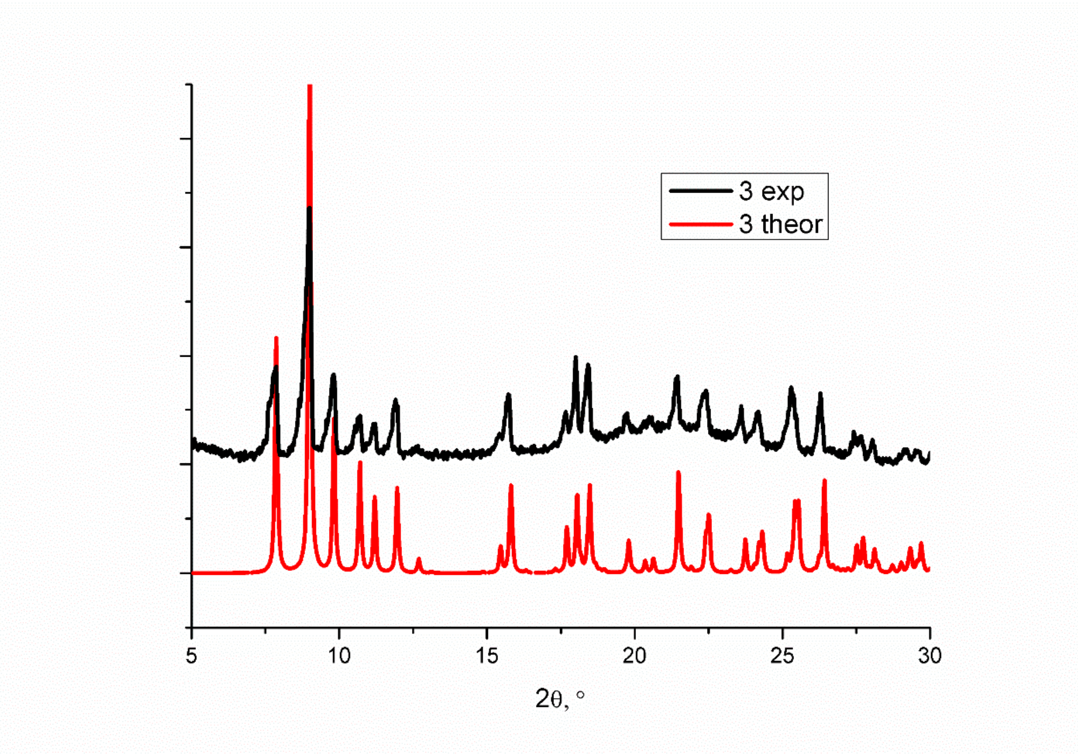

Figure S2. PXRD pattern of the synthesized sample of **3** (black) in comparison with the theoretical one (red).

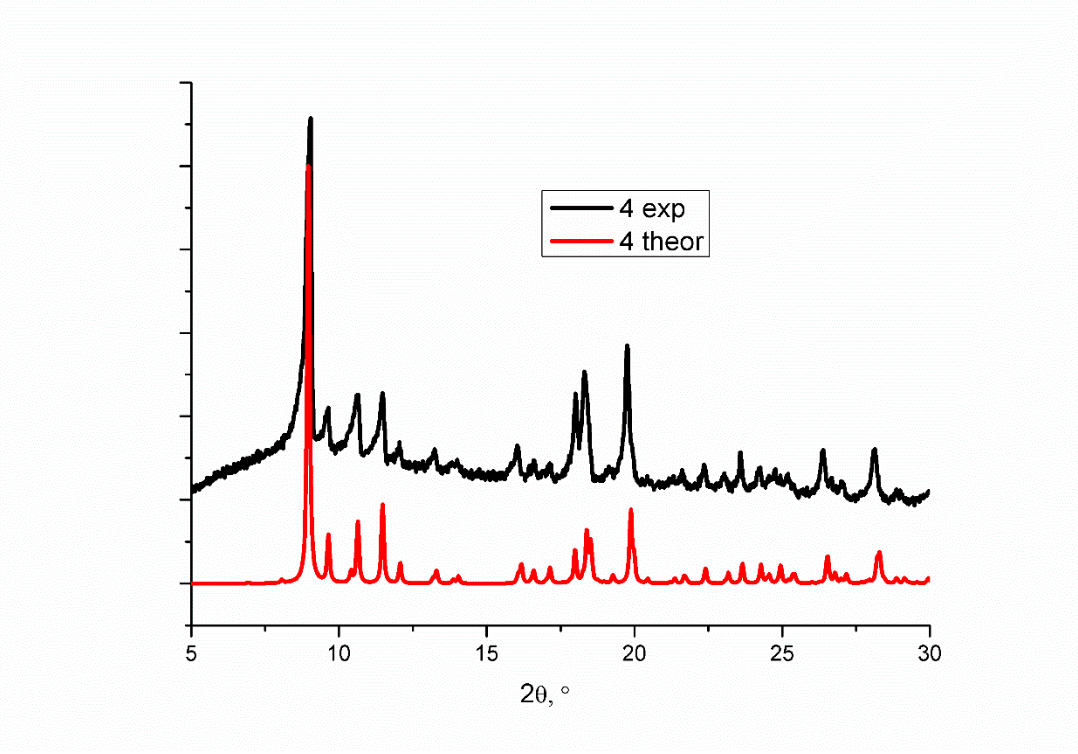

Figure S3. PXRD pattern of the synthesized sample of **4** (black) in comparison with the theoretical one (red).

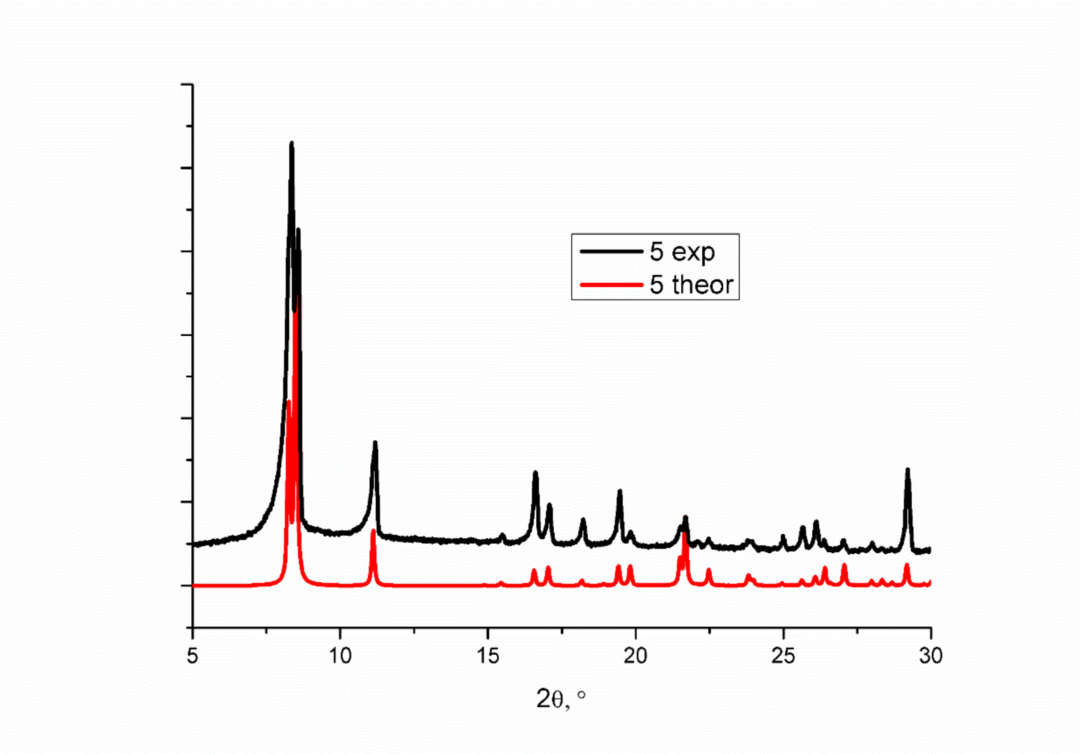

Figure S4. PXRD pattern of the synthesized sample of **5** (black) in comparison with the theoretical one (red).

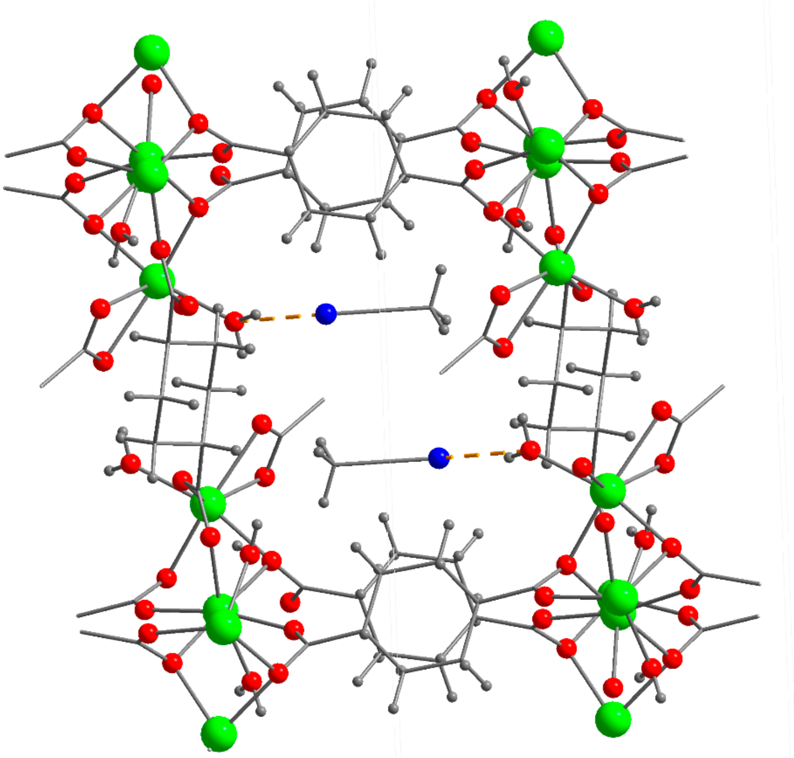

Figure S5. Location of guest CH_3CN molecules in the cage of **3**.

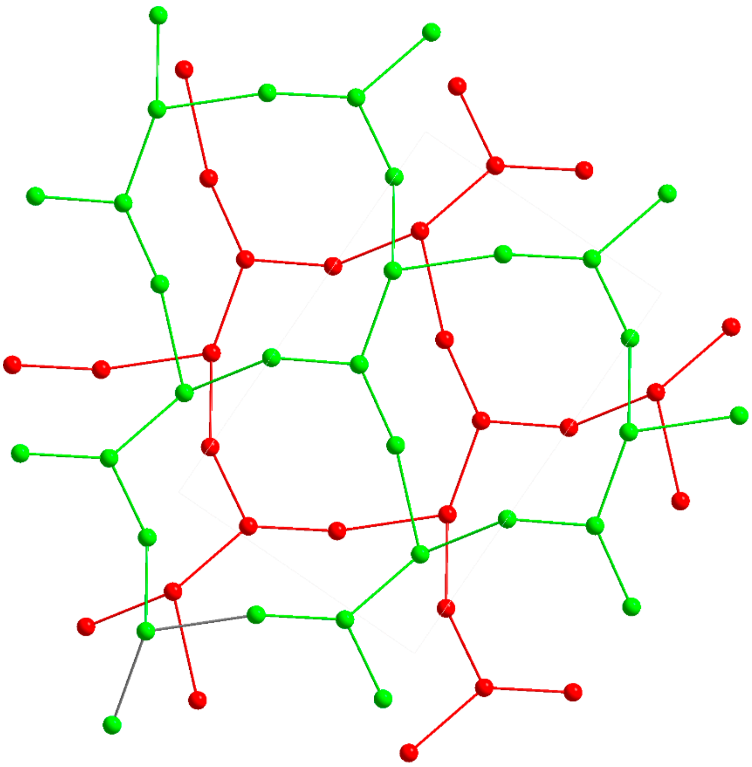

Figure S6. Interconnection between **AB** layers into 3D framework in **4**.

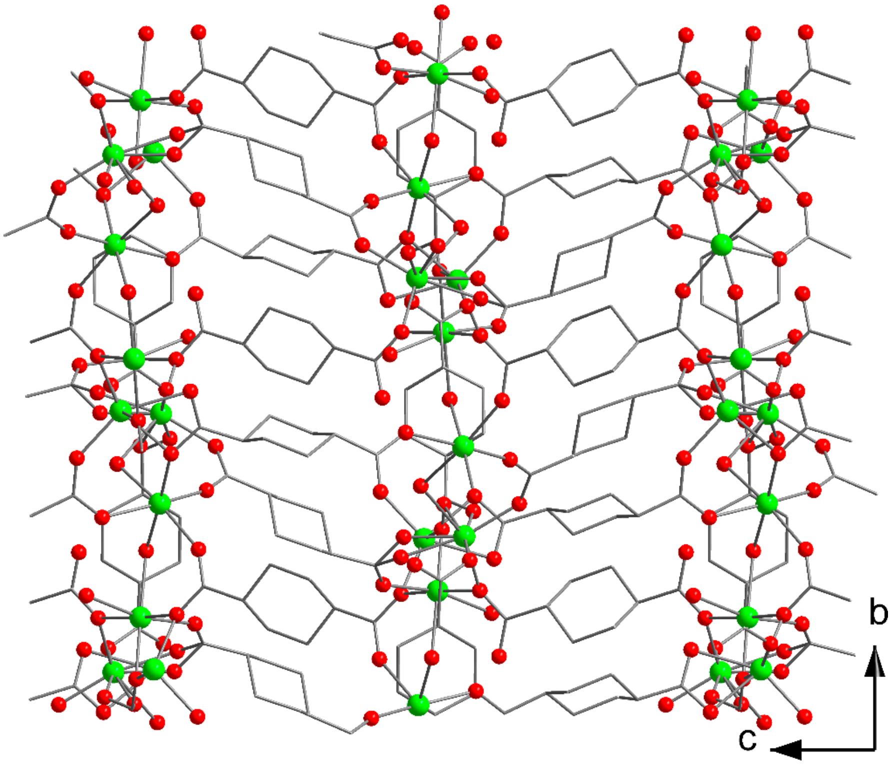

Figure S7. The schematic illustration of AB-packing in **4**.

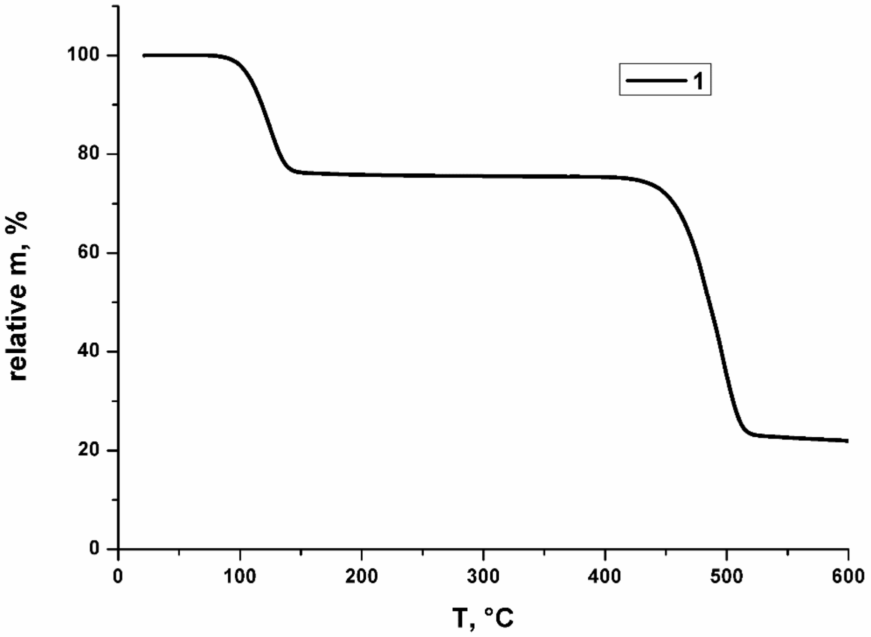

Figure S8. TG plot for the compound **1**.

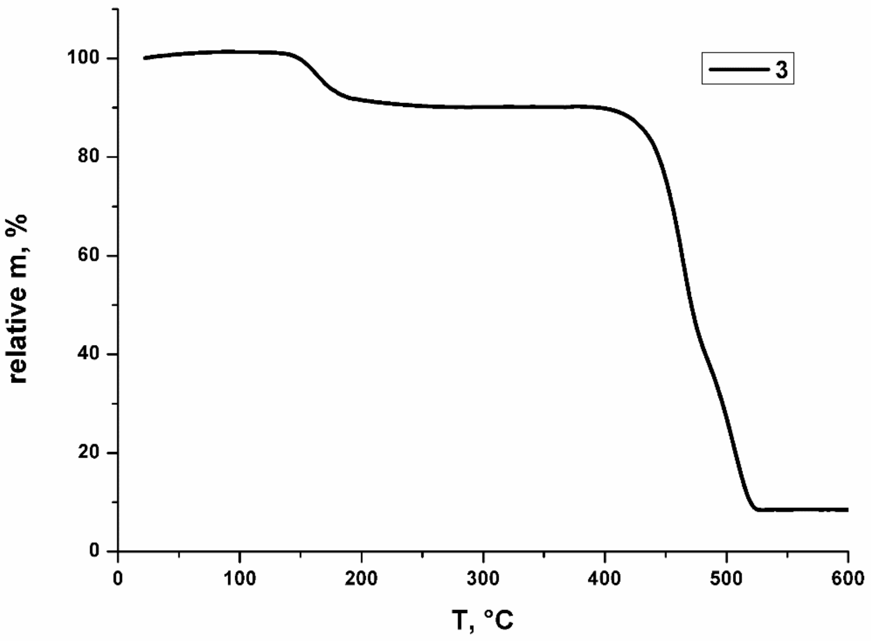

Figure S9. TG plot for the compound **3**.

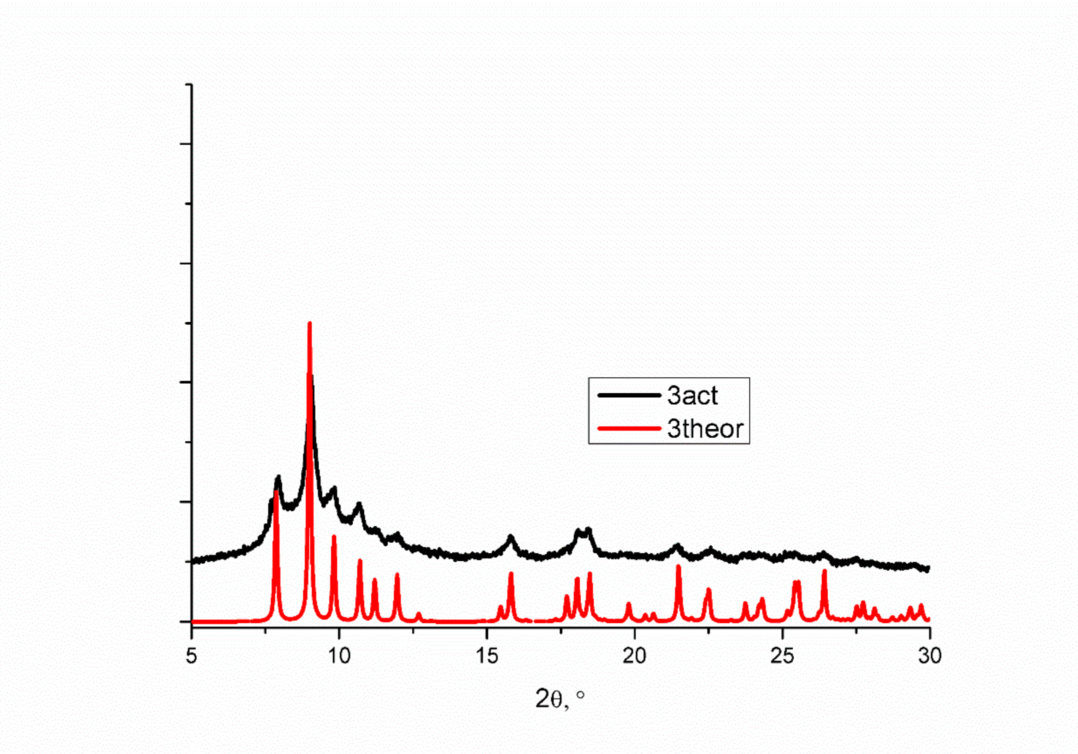

Figure S10. PXRD pattern of the activated sample of **3** (black) in comparison with the theoretical for **3** (red).

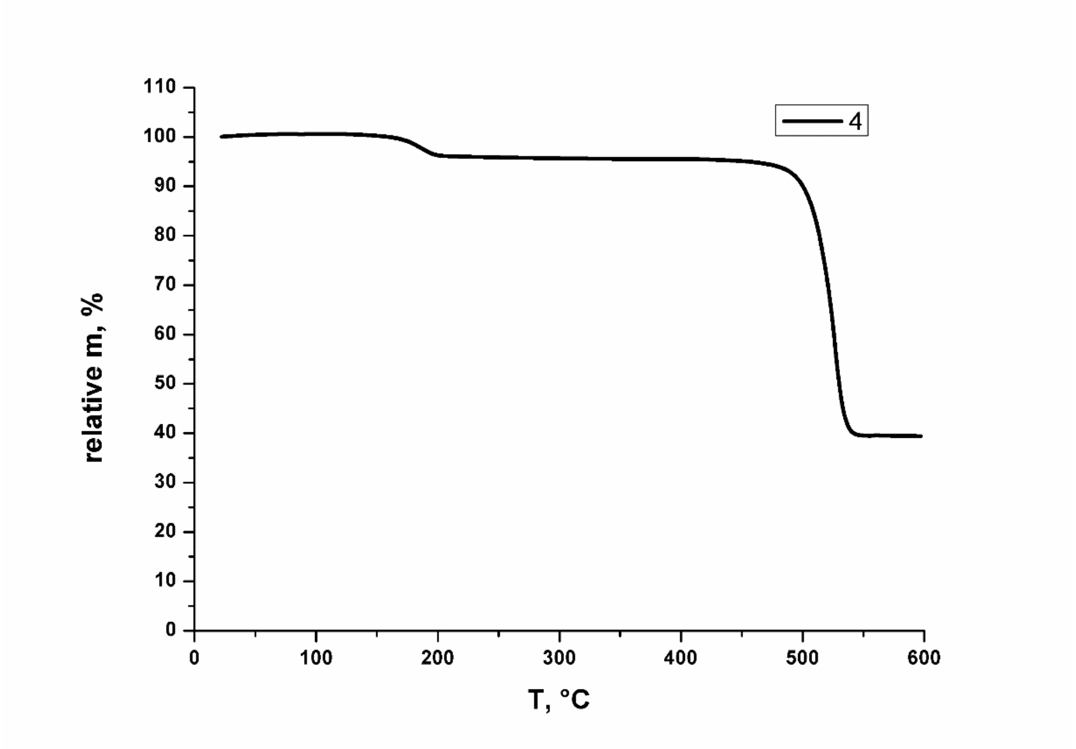

Figure S11. TG plot for the compound **4**.

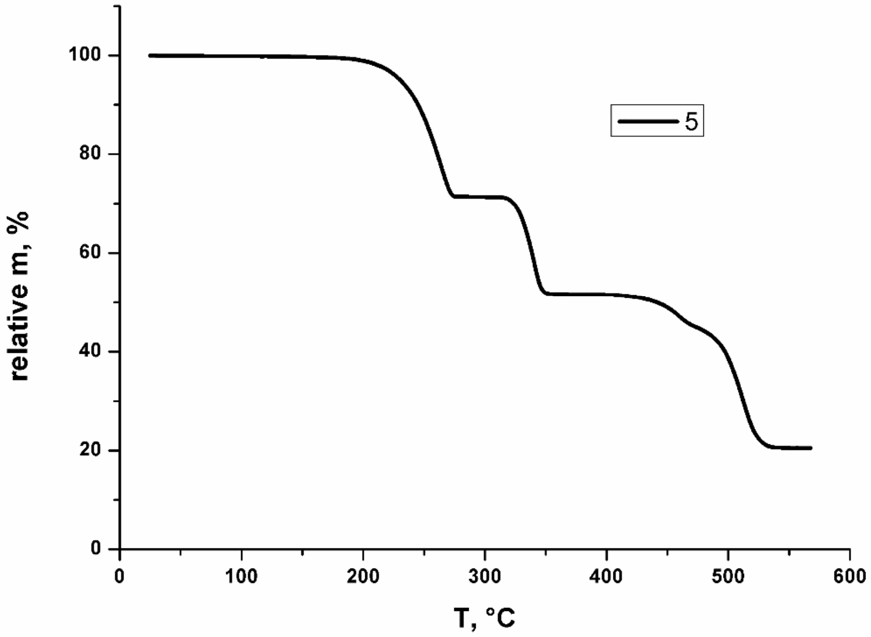

Figure S12. TG plot for the compound **5**.

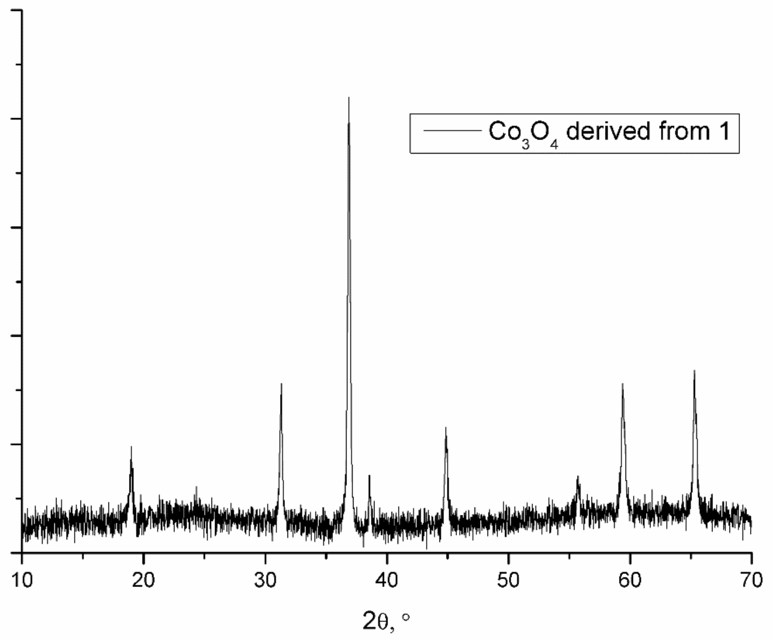

Figure S13. PXRD pattern of the Co_3O_4 sample derived from **1** by the oxidative thermolysis.

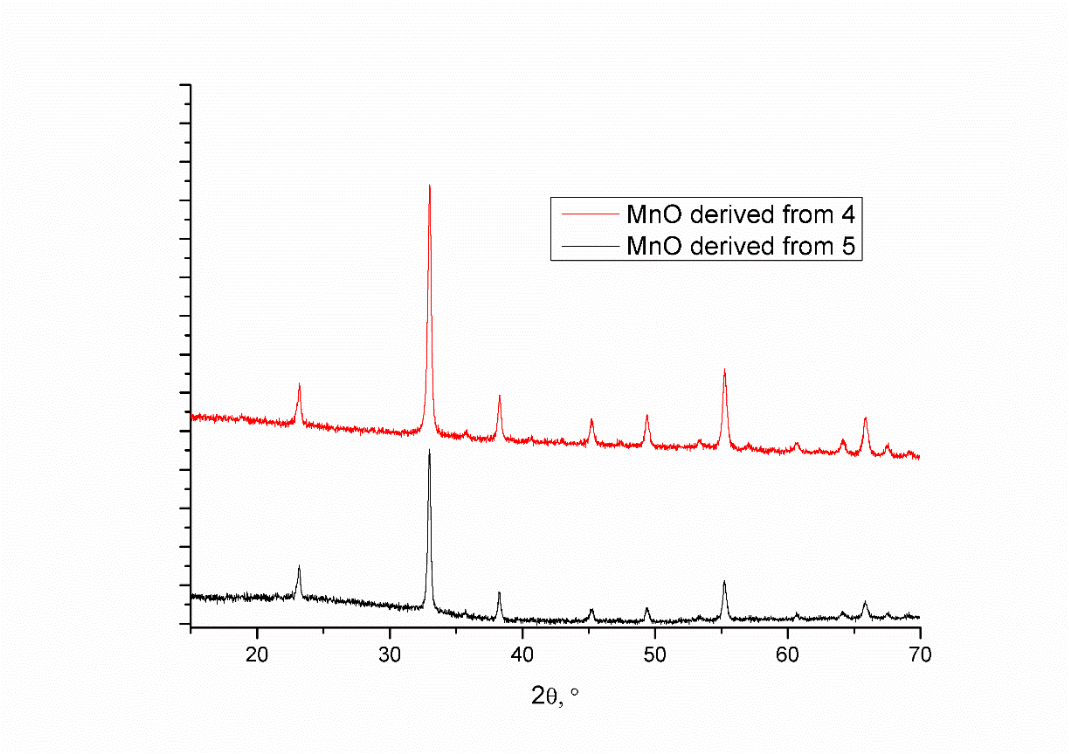

Figure S14. PXRD patterns of the MnO samples derived from **4** (red) and **5** (black) by the oxidative thermolysis.

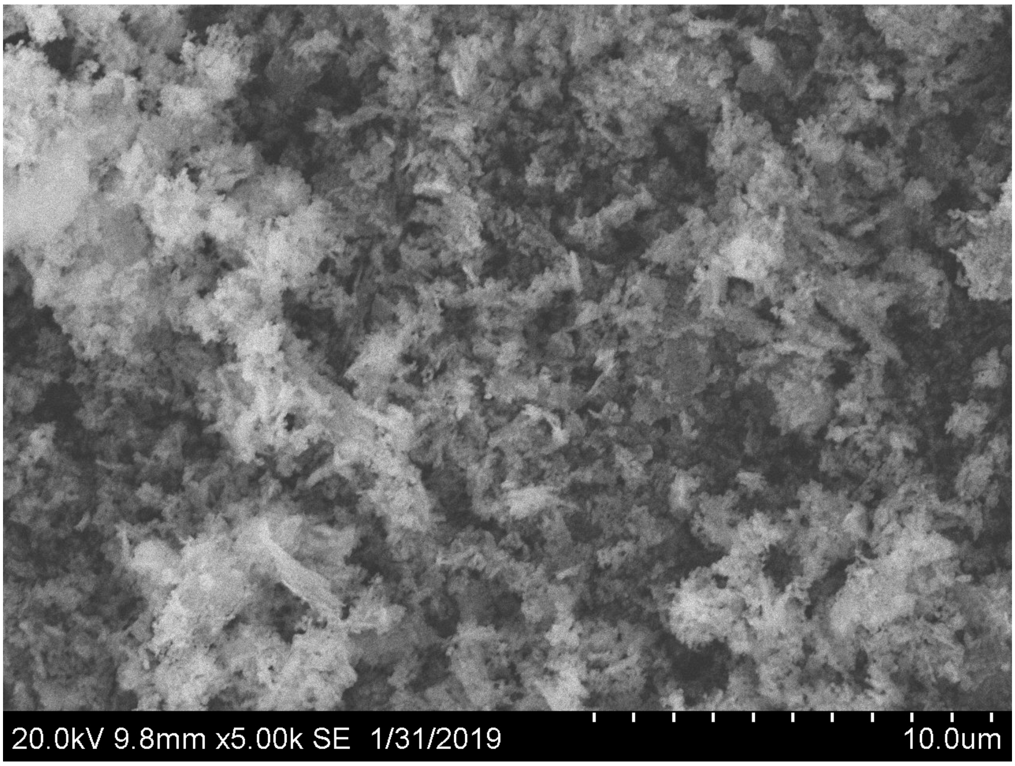

Figure S15. SEM image of Co_3O_4 sample obtained by the oxidative thermolysis of **1**.

© 2020 by the authors. Licensee MDPI, Basel, Switzerland. This article is an open access article distributed under the terms and conditions of the Creative Commons Attribution (CC BY) license (http://creativecommons.org/licenses/by/4.0/).
